# A Novel Deep Neural Network Model for Multi-Label Chronic Disease Prediction

**DOI:** 10.3389/fgene.2019.00351

**Published:** 2019-04-24

**Authors:** Xiaoqing Zhang, Hongling Zhao, Shuo Zhang, Runzhi Li

**Affiliations:** Collaborative Innovation Center of Internet Healthcare, Zhengzhou University, Zhengzhou, China

**Keywords:** multi-label classification, chronic disease, group block, GroupNet, correlated loss

## Abstract

Chronic diseases are one of the biggest threats to human life. It is clinically significant to predict the chronic disease prior to diagnosis time and take effective therapy as early as possible. In this work, we use problem transform methods to convert the chronic diseases prediction into a multi-label classification problem and propose a novel convolutional neural network (CNN) architecture named GroupNet to solve the multi-label chronic disease classification problem. Binary Relevance (BR) and Label Powerset (LP) methods are adopted to transform multiple chronic disease labels. We present the correlated loss as the loss function used in the GroupNet, which integrates the correlation coefficient between different diseases. The experiments are conducted on the physical examination datasets collected from a local medical center. In the experiments, we compare GroupNet with other methods and models. GroupNet outperforms others and achieves the best accuracy of 81.13%.

## Introduction

Chronic diseases account for a majority of healthcare costs and they have been the main cause of mortality in the worldwide (Lehnert et al., 2011; Shanthi et al., 2015). With the development of preventive medicine, it is very important to predict chronic diseases as early as possible. However, it is difficult for clinicians to make useful diagnosis in advance, because the pathogeny of chronic disease is fugacious and complex. In general, clinicians firstly form the diagnostic results of chronic disease according to the physical examination records based on their expertise and experience. Nevertheless, with more and more physical examination records produced, clinicians would have difficulty forming accurate diagnosis in limited time. Artificial intelligence technology has brought enormous reform in medical domain, and it can help doctor diagnose by forming the diagnostic results automatically based on the prediction models. In clinical practice, a symptom is always associated with multiple chronic diseases based on the physical examination records. Hence, the diagnosis or prediction of multiple chronic diseases could be transformed into a multi-label classification problem.

Multi-label classification problem is one of the supervised learning problems where an instance may be associated with multiple labels simultaneously. Currently, Multi-label classification problems have appeared in more and more applications, such as diseases prediction, semantic analysis, object tracking, and image classification, etc. Many successful multi-label algorithms have been obtained by the problem transformation methods. Problem transformation methods firstly convert the multi-label classification problems into several binary classification problems or a multi-class classification problem, and then apply original machine learning algorithms to handle them. The binary relevance (BR) method and label powerset (LP) method (Zhang and Zhou, 2014) are two representative label transformation methods. Plenty of competitive machine learning algorithms have been proposed based on problem transformation methods in the literatures, such as support vector machines (SVM) (Gu et al., 2015; Khan et al., 2018), decision tree (DT) (Hong et al., 2018), random forest (RF) (Murphy, 2018), etc.

Currently, deep learning technique is applied to various fields successfully since it provides a more efficient learning mechanism for classification problems than classical machine learning methods. For medical data analysis, numerous machine learning methods have been applied to analyze various medical data. BPMLL (Zhang and Zhou, 2006) is a back-propagation neural network for multi-label functional genomics classification, and it addresses correctly predicted labels that should be ranked higher than those mistakenly predicted labels by modifying the loss function. Lipton et al. (2015) utilized the LSTM to analyze time-series clinical data to diagnose 128 different diseases. In order to reduce over-fitting and improve the classification performance of the LSTM architecture, label replication and auxiliary outputs strategies were applied in their work. Maxwell et al. (2017) used a 2-layer deep neural network to classify three chronic diseases based on physical examination records and found combine deep learning algorithms with RAkEL (Tsoumakas and Vlahavas, 2007) method that could improve multi-label classification performance. Miotto et al. (2016) combined a 3-layer autoencoder (AE) and logistic regression classifiers to predict ICD 9-based disease diagnosis using a prediction window. Liang et al. (2014) used a Deep Belief Network (DBN) to generate patient vectors, and then applied a support vector machine (SVM) to classify these generated patient vectors for general disease diagnoses. Jin et al. (2018) made hospital mortality prediction with medical named entities and multimodal learning based on the Long Short-Term Memory (LSTM) architecture, and they outperformed the benchmark by 2% AUC. However, applying deep learning technique to the medical data is still challenging because medical data are sparse, heterogeneous and unstructured.

In this work, we apply the convolutional neural network (CNN) to handle the classification of multiple chronic diseases based on the physical examination records. Because the CNN is the most widely used deep learning method, and it usually gets the desirable classification performance in various classification problems (such as medical image analysis, medical text analysis, and disease prediction). For multiple chronic diseases label transformation, we use two common problem transformation methods: binary relevance (BR) and label powerset (LP) methods in the data preprocessing phase, in order to get expected performance. BR converts multiple chronic disease classification problem into several binary chronic disease classification problems while LP transforms multiple chronic disease classification in a single-label multi-class classification problem.

The main contributions of this work can be summarized as following. Firstly, we devise the convolution block named group block, which both decreases the number of convolution parameter and enhances the overall classification performance. Secondly, a novel CNN architecture named GroupNet using group block is proposed for the classification of multiple chronic diseases based on the physical examination dataset. Thirdly, we devise the correlated loss (CL) to improve the classification performance used in the proposed GroupNet. The proposed GroupNet achieves the best accuracy of 81.13% and increases the overall classification results by at least 2.57% than any other state-of-art deep learning and machine learning methods.

The rest of this work is organized as follows. Section Dataset and Data Preprocessing introduces dataset and data preprocessing. Section Problem Formulation provides definition of the multi-label chronic disease prediction problem. The group convolution strategy, group block and GroupNet architecture are presented in Section Methods. Correlation loss and optimization strategies are elucidated in Section Loss Function and Optimization. Section Experiments and Evaluation describes experiment setup and evaluation measures. Results and Discussion are illuminated in Section Results and Discussion. Finally, Conclusions concludes this work along with future work.

## Dataset and Data Preprocessing

In the work, we mainly focus on multiple chronic disease classification. It can be formulated into a multi-label classification problem. There are three common chronic diseases are selected from the physical examination records: hypertension (H), diabetes (D), and fatty liver (FL).

In the experiments, the physical examination datasets are collected from a local medical center, which contain 110,300 physical examination records from about 80,000 anonymous patients (Li et al., 2017a,b). Sixty-two feature items are selected from over 100 examination items based on medical expert experience and related literature in every physical examination record. These feature items contain 4 basic physical examination items, 26 blood routine items, 12 urine routine items, and 20 items from liver function.

Two multi-label transformation methods consisting of binary relevance (BR) and label powerset (LP) method are used in this work. For BR method, the diagnosis of a given patient can be one of three possible results: all three chronic diseases, different combination of the chronic diseases, or no signs of any three chronic diseases, which means that there are totally eight different sets of diagnoses {000, 100, 010, 001, 110, 101, 011, 111}. Based on Label Powerset (LP) method, we get eight different prediction labels and can be represented by {0, 1, 2, 3, 4, 5, 6, 7}.

**Figure 1:**
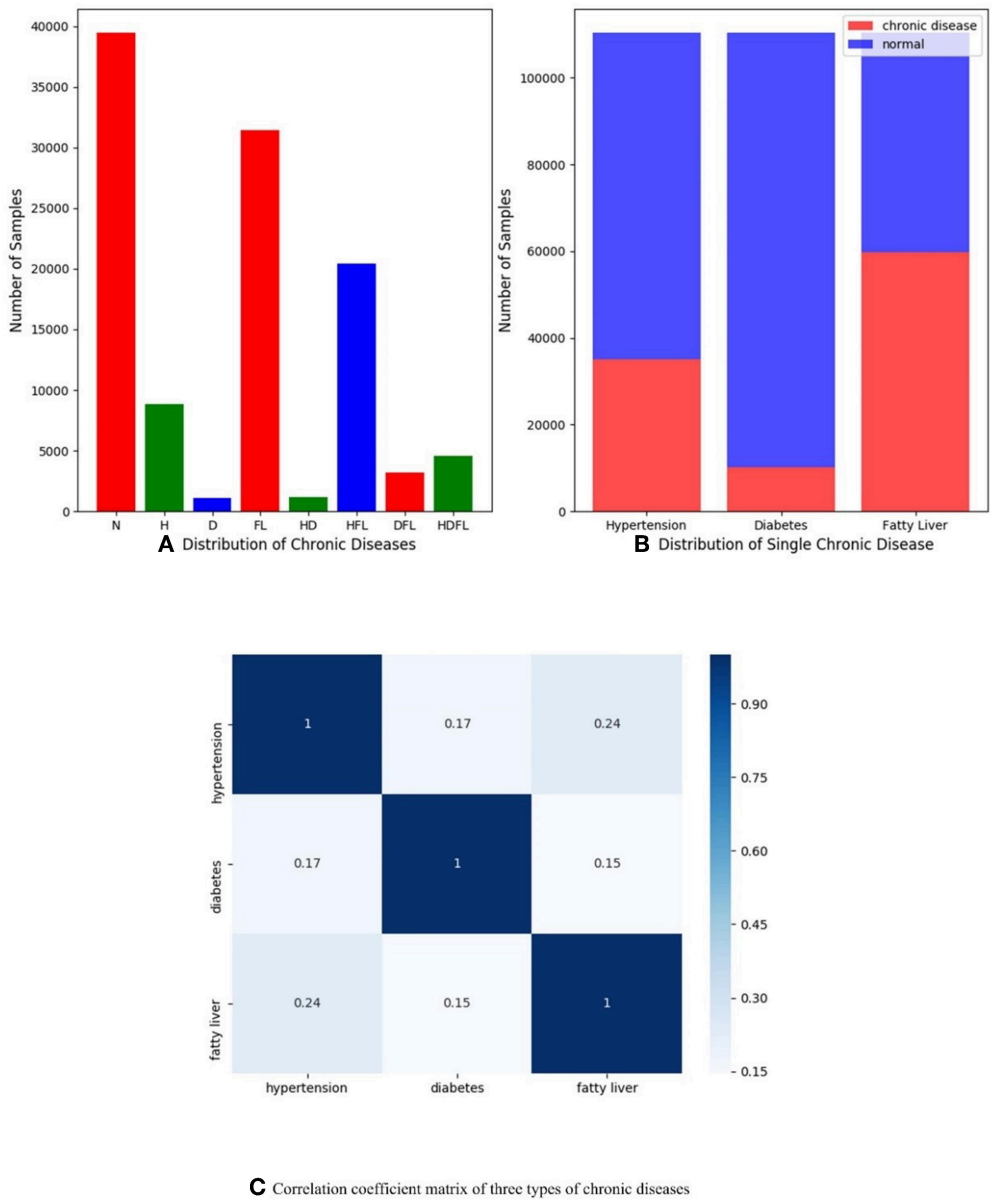
**(A)** Distribution of multiple chronic diseases; **(B)** Distribution of single-label of three chronic diseases dependencies; **(C)** Correlation coefficient matrix of three types of chronic diseases (hypertension, diabetes, and fatty liver), and they are computed by Pearson product-moment correlation coefficient.

In order to understand dataset better and receive expected results, we do some data analysis in the stage of data preprocessing as shown in Figure 1. Figure 1A presents the multi-label distribution of chronic diseases, and single-label distribution of three chronic diseases is shown in Figure 1B. The results demonstrate that the multi-label distribution of chronic diseases is highly skewed, 62.5% of physical examination records is occupied by normal and HFL, and while independent diabetes (D) only hold 1% of physical examination records according to Figure 1A. The single label distribution of fatty liver is a balanced proportion, while the single label distributions of hypertension and diabetes are both imbalanced as you can see from Figure 1B. The correlation coefficient analysis can indicate the label dependencies, and it can be calculated by Pearson product-moment correlation coefficient (PMMC) (Mohamad Asri et al., 2018; Weber and Immink, 2018). Figure 1C shows that the correlation coefficient value between hypertension and flatty liver is maximum among three chronic disease pairs, but the correlation coefficient value is only 0.24. According to the theory of correlation coefficient, we can infer that the correlation between three chronic diseases are not strong.

We firstly use simple data augmentation method to handle label imbalance problem. However, this method does not work as we expected likely due to the fact that correlation coefficient value among diseases is small as you can see in Figure 1C. Focal loss (Lin et al., 2017) strategy is utilized to relieve label imbalance problem in this work. Furthermore, a cost-sensitive loss learning algorithm called correlated loss (CL) would be described in Group Convolution Strategy in detail and correlation coefficient values between chronic diseases is used as hyper-parameters in the correlated loss. The correlation loss is mainly proposed for improving overall classification performance. Physical examination data are split into two parts, 70% of the data for training and 30% of the data for testing in the experiments.

## Problem Formulation

In medicine filed, the goal of multiple chronic diseases prediction is to predict onset of chronic diseases in advance based on disease prediction model. To this end, we solve multiple chronic diseases prediction problem based on the physical examination dataset. It can be formulated into a multi-label classification problem in computer science. Firstly, we use problem transform methods to transform multiple chronic disease classification problem into multi-label problem classification. Secondly we construct CNN architectures to resolve the multi-label classification.

## Methods

### Group Convolution Strategy

To improve the performance of a convolutional neural network (CNN) architecture. It is easy to be adopted that we increase the number of convolution kernel in every convolution layer simply. However, it would increase the number of convolution parameter and weaken the classification results. Some well-known and successful convolutional neural network architectures have been proposed to handle this problem, such as IGCNets (Zhang et al., 2017; Sun et al., 2018; Xie et al., 2018), and ShuffleNet (Ma et al., 2018). One common ground for these CNN architectures is that they are implemented based on group convolution strategy (Krizhevsky et al., 2012).

**Figure 2:**
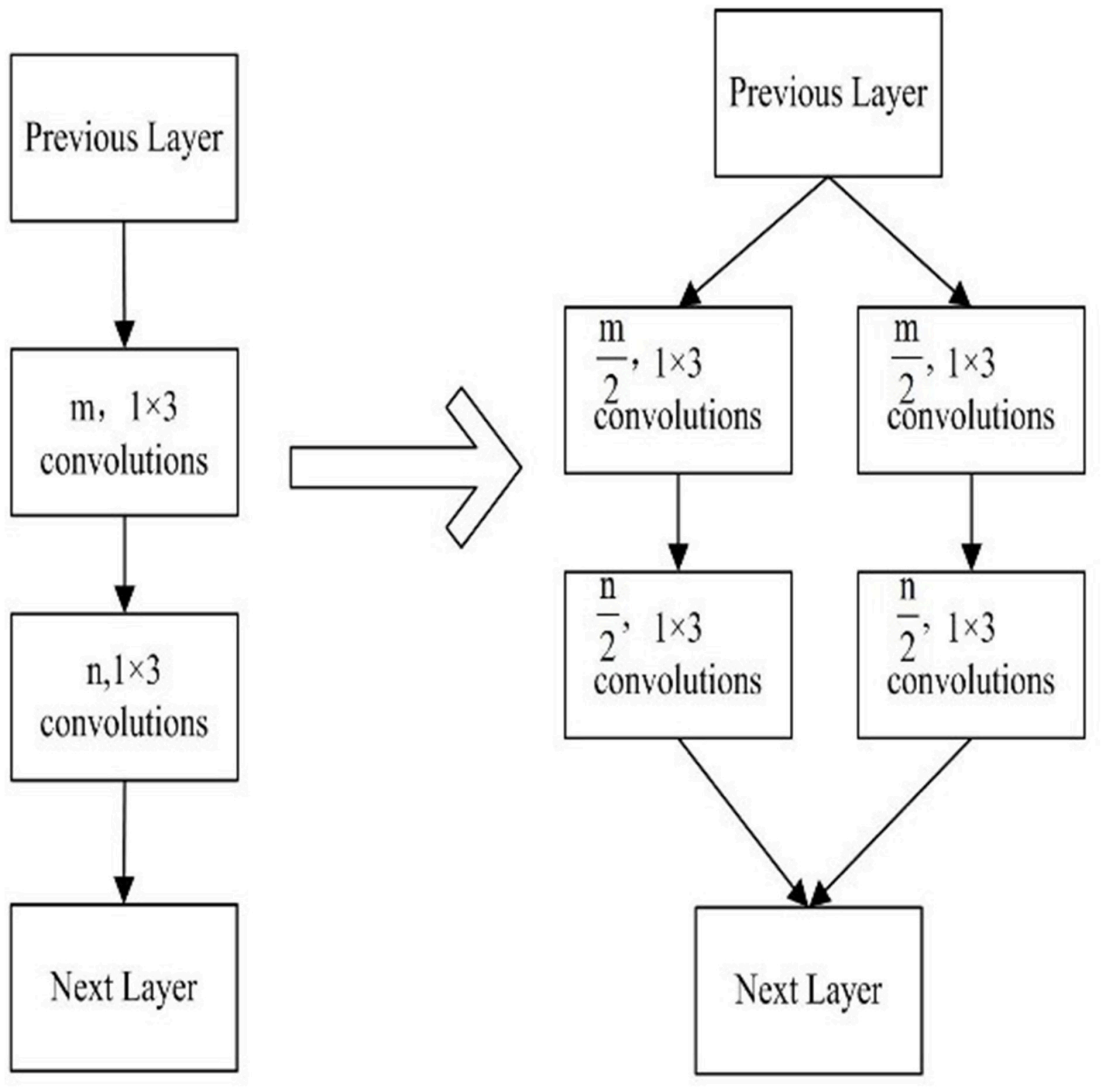
Group convolution strategy.

In the implementation of the group convolution strategy, there are being two continuous convolution layers at least. The number of convolution kernel in every convolution layer is split into several independent group convolution partitions. An example of group convolution strategy is shown in Figure 2. A CNN model consists of two continuous convolution layers, in which m and n convolution kernels are set respectively. By the group convolution strategy, we split every convolution layer into two partition convolution units and the number of convolution kernels is the half. The reduction of convolution parameters is shown in Equation 1.

(1) $$\begin{array}{l} \frac{2\times \frac{m}{2}\times \frac{n}{2}\times 1\times 3}{m\times n\times 1\times 3}=\frac{1}{2} \end{array}$$

### Group Block

**Figure 3:**
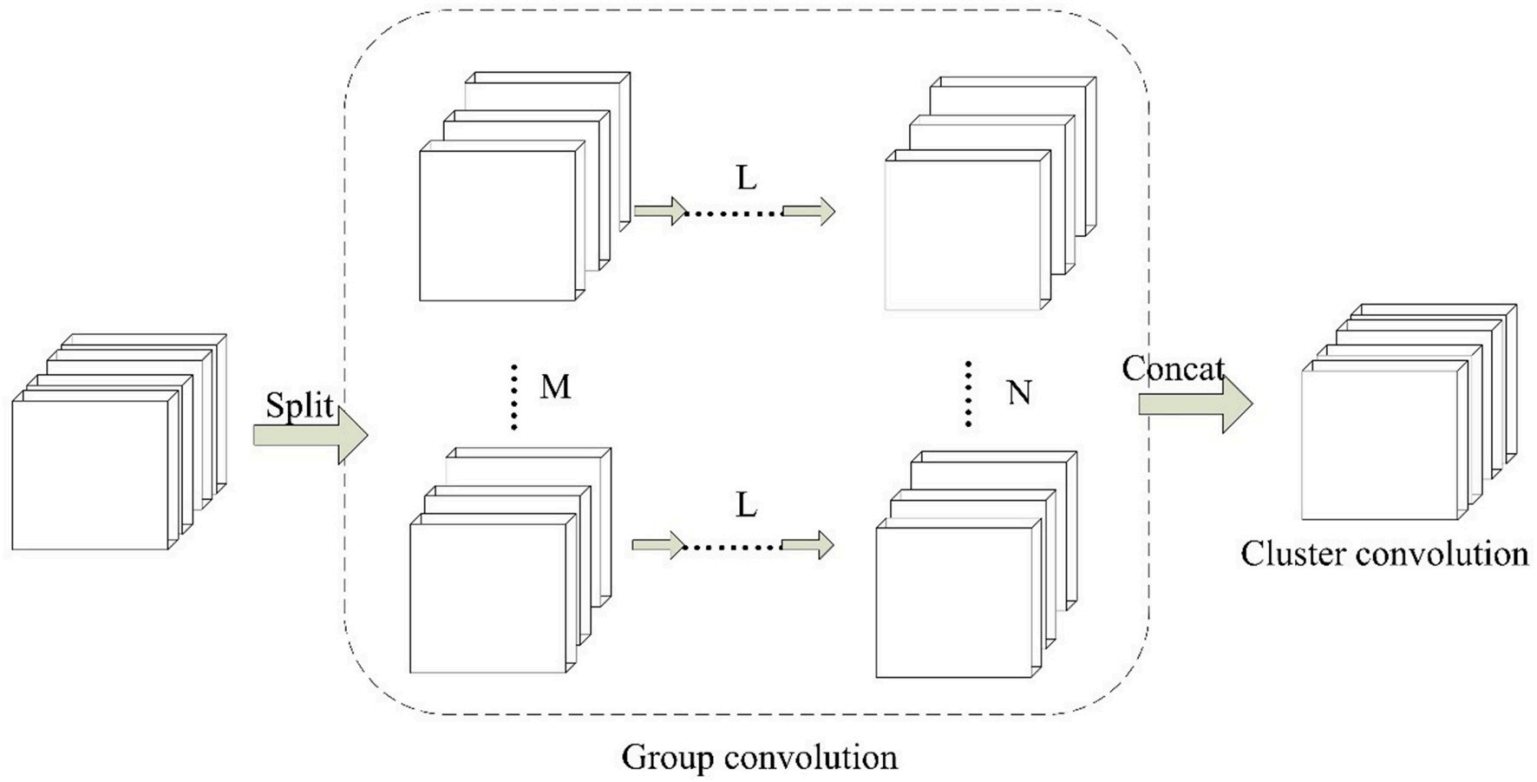
The paradigm of Group Block. For L continuous convolution layers, M and N denotes the number of independent partition convolution units.

Inspired by group convolution strategy, we propose the group block in this work. Group block consists of two parts, which are group convolution and cluster convolution. The architecture of group block is shown in Figure 3.

In the group convolution part, it splits one convolution unit to multiple partition convolution units. The number of partition convolution units can be set randomly for different convolution layers L. For example, it can be set to split M or N convolution units. In cluster convolution part, a 1 × 1 convolution layer is designed after the group convolution part. It is implemented to cluster the correlated feature maps and enhances discriminability for local patches within the receptive field.

The parameters of group block are described by (L, N_i_ (i = 1, … m), j). Here L denotes the number of continuous convolution layers. N_i_ (i = 1, … m) shows the number of partition convolution units in the ith convolutional layer. j is the number of cluster convolution layers.

### GroupNet Architecture

**Figure 4:**
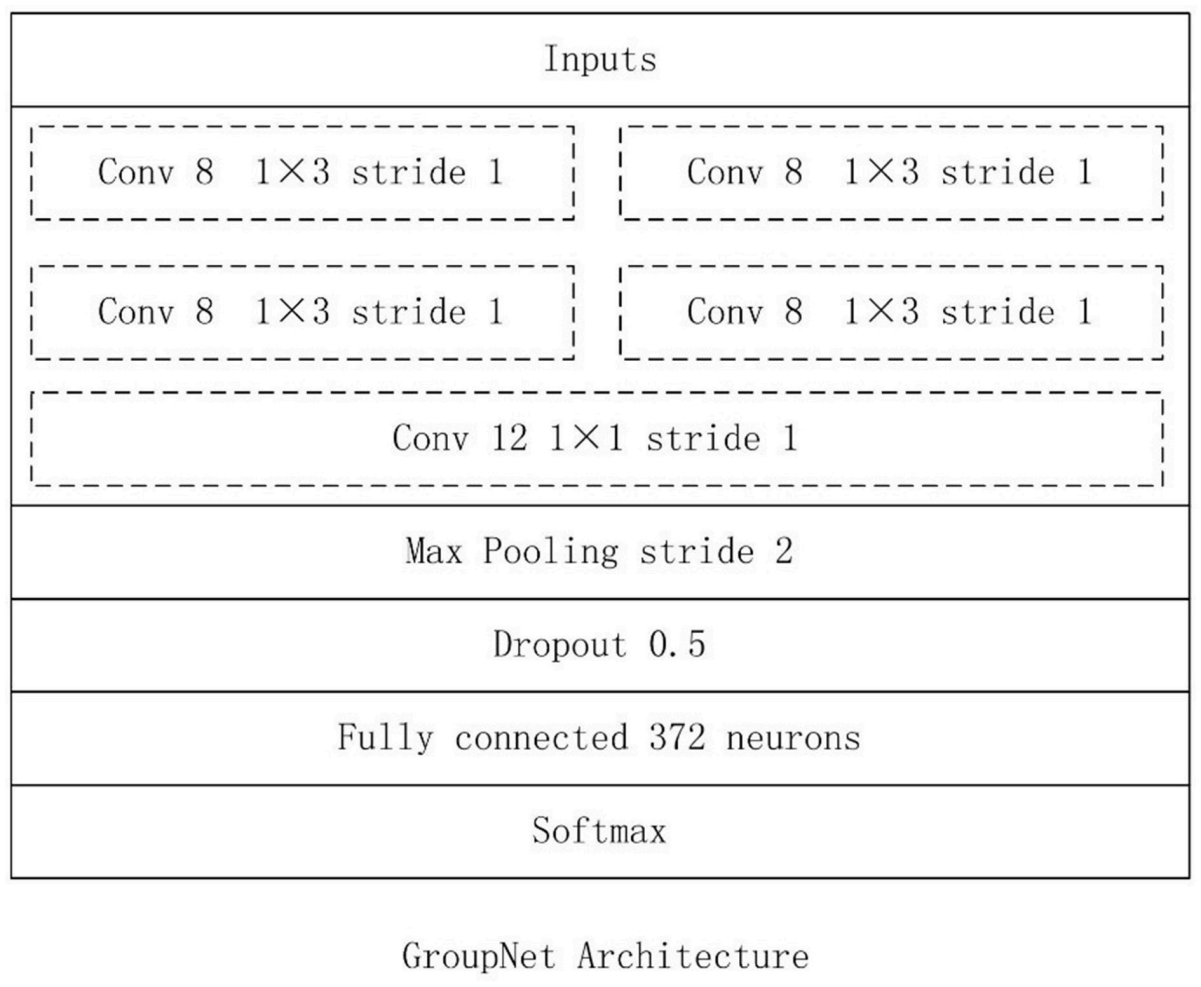
GroupNet Architecture.

In this work, we construct the CNN architecture based on the proposed group block named the GroupNet, shown in Figure 4. The proposed group block is the core part of the GroupNet, which is a variant of group convolution. The main difference between the proposed group block and the traditional group convolution is that we add a cluster convolution part after group convolution part in group block. Hence, the GroupNet architecture built on the group block improves the classification performance efficiently when comparing to several advanced CNN architectures.

**Figure 5:**
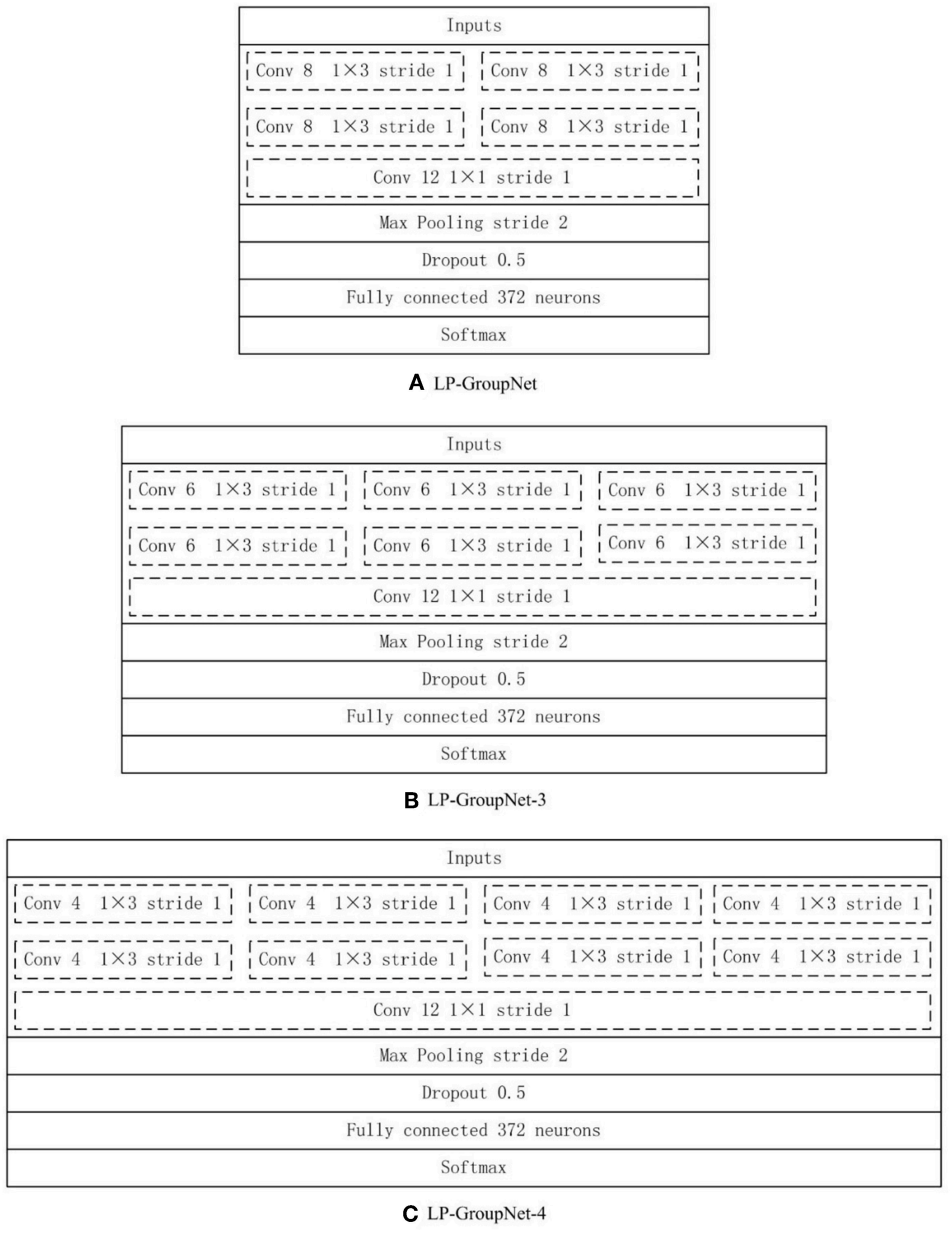
**(A)** LP-GroupNet, **(B)** LP-GroupNet-3, **(C)** LP-GroupNet-4.

The GroupNet architecture contains six layers: input layer, group block, max-pooling layer, dropout layer, fully-connected layer and softmax layer. The detail parameters of GroupNet architecture is listed in Figure 5. Small convolution kernels always are used to reduce the computation burden and improve the classification performance (Huang et al., 2016; Iandola et al., 2016; Sandler et al., 2018). In this work, we use 1 × 3 as the convolution kernel size. Because convolution kernel size 1 × 3 achieves better performance than other convolution kernel sizes in the experiments. Because physical examination data are one-dimensional. Hence, one-dimensional convolution kernel is adopted. Furthermore, softmax function is used as classifier, because it is standard to use the softmax as classifier in deep learning.

Well-known dropout (Srivastava et al., 2014; Bouthillier et al., 2015) technique is available to alleviate over-fitting for CNN. In this work, we set a dropout layer between the max-pooling layer and the fully-connected layer and the drop rate is 0.5 which is set experimentally.

In this work, LP and BP are adopted to resolve the multi-label classification, respectively. LP method is to transform multiple chronic disease classification into the single-label multi-class classification, while BR method converts the multi-label chronic disease classification into three binary classifications. Correspondingly, LP-GroupNet and BR-GroupNet are named in experiments.

## Loss Function and Optimization

### Correlated Loss

Focal loss (FL) (Lin et al., 2017) is a variant of standard cross entropy loss, and it alleviates loss of correctly classified examples domain the gradient in the training and can be computed as following.

(2) $$\begin{array}{l} FL\left(p\right)=-{\left(1-p\right)}^{\gamma }\log p \end{array}$$

Here p is the probability for predicted label. (1 − *p*)^γ^ is modulating factor and γ is a focusing parameter. γ is set manually. When γ = 0, focal loss is equal to standard cross entropy loss. The cross entropy loss is described as following.

(3) $$\begin{array}{l} CE\left(p,q\right)=-q\log p \end{array}$$

CE (p, q) is a cross entropy loss, *p* and *q* represent the expected output and actual output, respectively.

In the BR-GroupNet architecture, each binary classifier is independent of each other, in order to enhance connection between independent classifiers and each classifier can learn useful information from each other. Hence, we propose a cost-sensitive learning algorithm named correlated loss (CL) for the BR-GroupNet to enhance classification performance by learning loss information from each other. In the BR-GroupNet architecture, the correlated loss of each binary classifier consists of two parts: main loss and auxiliary loss. Main loss can be computed by the classifier itself and auxiliary loss is the sum of product associated classifier loss and correlation coefficient value. In this work, correlation coefficient value between two chronic diseases is chosen as a hyper parameter in auxiliary loss, because correlation coefficient value between two diseases is small and it also indicates disease dependencies between two diseases. Therefore, correlated loss (CL) of an independent binary classifier in BR-GroupNet can be computed as follows.

(4) $$\begin{array}{l} CL=loss+\sum_{i=1}^{2}\alpha_{i}loss_{i} \end{array}$$

(5) $$\begin{array}{l} CL1=CE+\sum_{i=1}^{2}\alpha_{i}CE_{i} \end{array}$$

(6) $$\begin{array}{l} CL2=FL+\sum_{i=1}^{2}\alpha_{i}FL_{i} \end{array}$$

Here α is a correlation coefficient value between every two labels, which is calculated by Pearson product-moment correlation coefficient (PMMC). In this work, we choose three chronic diseases as multi-label chronic disease prediction targets and only three independent binary classifiers are required. For the correlated loss of each independent classifier, the loss of each classifier itself as main loss, and the sum of product of two associated classifier losses and correlation coefficient values as the auxiliary loss. Hence, we set the range of parameter i from 1 to 2 in this work.

In this work, we use two different methods to calculate the correlated loss based on CE and FL, respectively, and named CL1 and CL2 as seen in Equations 5, 6. In order to validate whether selecting correlation coefficient value between two chronic diseases as hyper parameters of CL can work as we expected. The GroupNet architecture with correlated loss named BR-GroupNet-CL.

### Optimization

In the training of CNN models, back-propagation method is carried out for the gradient. There are many hyper parameters of CNN models that need to be optimized. It is experimental, time-consuming and difficult to choose best hyper parameters. To initialize hyper parameters with less tuning in the training phase, Adam (Kingma and Ba, 2014; Chen et al., 2018; Reddi et al., 2018) optimizer is used for the gradient. It is a first-order gradient-based descent optimizer of stochastic objective function. Adam is based on adaptive estimates of lower-order moments and computes individual learning rates for different hyper parameter from estimates of first and second moments of the gradients. Comparing to stochastic gradient descent optimization (SGD) (Orr and Müller, 2003), Adam is more efficient, which requires less memory and training time.

The proper activation function also improves classification performance. There are several popular activation functions for neural networks, such as sigmoid, tanh, rectified linear unit (ReLU) (Nair and Hinton, 2010), Leaky ReLU (LeakyReLU) (Maas et al., 2013), Exponential Linear Units (ELU) (Clevert et al., 2015), Self-Normalizing Linear Units (SELU) (Klambauer et al., 2017), and so on. In this work, we test and compare all different activation functions in our datasets and choose the preferable one in all CNN models.

## Experiments and Evaluation

### Experiment Setup

We implement all experiments based on the Scikit-learn library, WEKA software and Tensorflow platform. Scikit-learn library and WEKA are used to implement several machine learning methods, such as SVM, SMO, DT, Multilayer Perceptron (MLP). Tensorflow platform is used to implement deep learning methods, such as the proposed GroupNet architectures, IGCNet, GoogleNet (Szegedy et al., 2015), VGGNet (Simonyan and Zisserman, 2014), AlexNet, and deep neural network (DNN), Long Short-Term Memory (LSTM), and Gated Recurrent Unit (GRU) (Shickel et al., 2018). The experiments run on a machine with Intel (R) 3.20 GHz CPU (i5-6500) and 8 GB RAM.

Furthermore, several experiments are conducted to select proper parameters based on the LP-GroupNet, such as batch size, learning rate, epochs, convolution kernel size, dropout rate, activation function, and focusing parameter γ in focal loss. In order to select preferable number of convolution units of group block for the GroupNet, we deploy three GroupNet architectures based on three different group blocks. The detail parameter setting of three different group blocks are {2, 2, 2, 1}, {2, 3, 3, 1} and {2, 4, 4, 1}, and Figure 5 gives concrete CNN architectures of the three different GroupNet architectures, namely LP-GroupNet (Figure 5A), LP-GroupNet-3 (Figure 5B), and LP-GroupNet-4 (Figure 5C).

### Evaluation Measures

Since multi-label classification can be converted into single-label multi-class classification and so the measures to evaluate single-label multi-class classification also can be used for this work. We adopt four common evaluation measures: F-score, accuracy, recall and precision measures to compare the performance of different methods for multi-label chronic disease classification. The accuracy is a measure to ensure that ratio of the prediction of true labels is correct. Precision is a measure system that is related to reproducibility, or how many predictions are correct. Recall is the fraction of true labels that were predicted correctly. F-score (F1) measure is the harmonic mean of precision and recall, and is a popular evaluation measure in the research area of data mining. Because the label distribution of chronic disease is skewed as described in Dataset and Data Preprocessing, weighted recall, weighted precision, weighted F-score are used to evaluate the classification performance of different methods. F1 evaluates the overall performance of the method better than accuracy, precision and recall according to related works (Tsoumakas and Katakis, 2007; Zhang and Zhou, 2014). Recall is an important evaluation measure in clinical. Different to normal F-score, the value of weighted F-score is not between weighted precision and weighed recall, instead it is smaller than both weighted precision and weighed recall. The following equations show how to calculate these values. TP, TN, FP, and FN are true positive, true negative, false positive, and false negative, respectively.

(7) $$\begin{array}{lll} Accuracy & = & \frac{\sum_{i=1}^{l}\frac{TP_{i}+TN_{i}}{TP_{i}+FP_{i}+TN_{i}+FN_{i}}}{l} \end{array}$$

(8) $$\begin{array}{lll} Precision_{weighted} & = & \sum_{i=1}^{l}k_{i}\frac{TP_{i}}{TP_{i}+FP_{i}} \end{array}$$

(9) $$\begin{array}{lll} Recall_{weighted} & = & \sum_{i=1}^{l}k_{i}\frac{TP_{i}}{TP_{i}+FN_{i}} \end{array}$$

(10) $$\begin{array}{l} F1_{weighted}=\sum_{i=1}^{l}k_{i}\frac{\frac{TP_{i}}{TP_{i}+FP_{i}}\cdot \frac{TP_{i}}{TP_{i}+FN_{i}}}{\frac{TP_{i}}{TP_{i}+FP_{i}}+\frac{TP_{i}}{TP_{i}+FN_{i}}}\\ \;=\sum_{i=1}^{l}k_{i}\frac{2Precision_{i}Recall_{i}}{Precision_{i}+Recall_{i}} \end{array}$$

Accuracy, *Precision*_*weighted*_, Re*call*_*weighted*_, and*F*1_*weighted*_can be computed by Equations (7–10). *k*_*i*_denotes the single labels accounted for the proportion of all labels, *l*is equal to 8 and i ranges 1–8.

## Results and Discussion

### Hyper Parameter Selection

**Figure 6:**
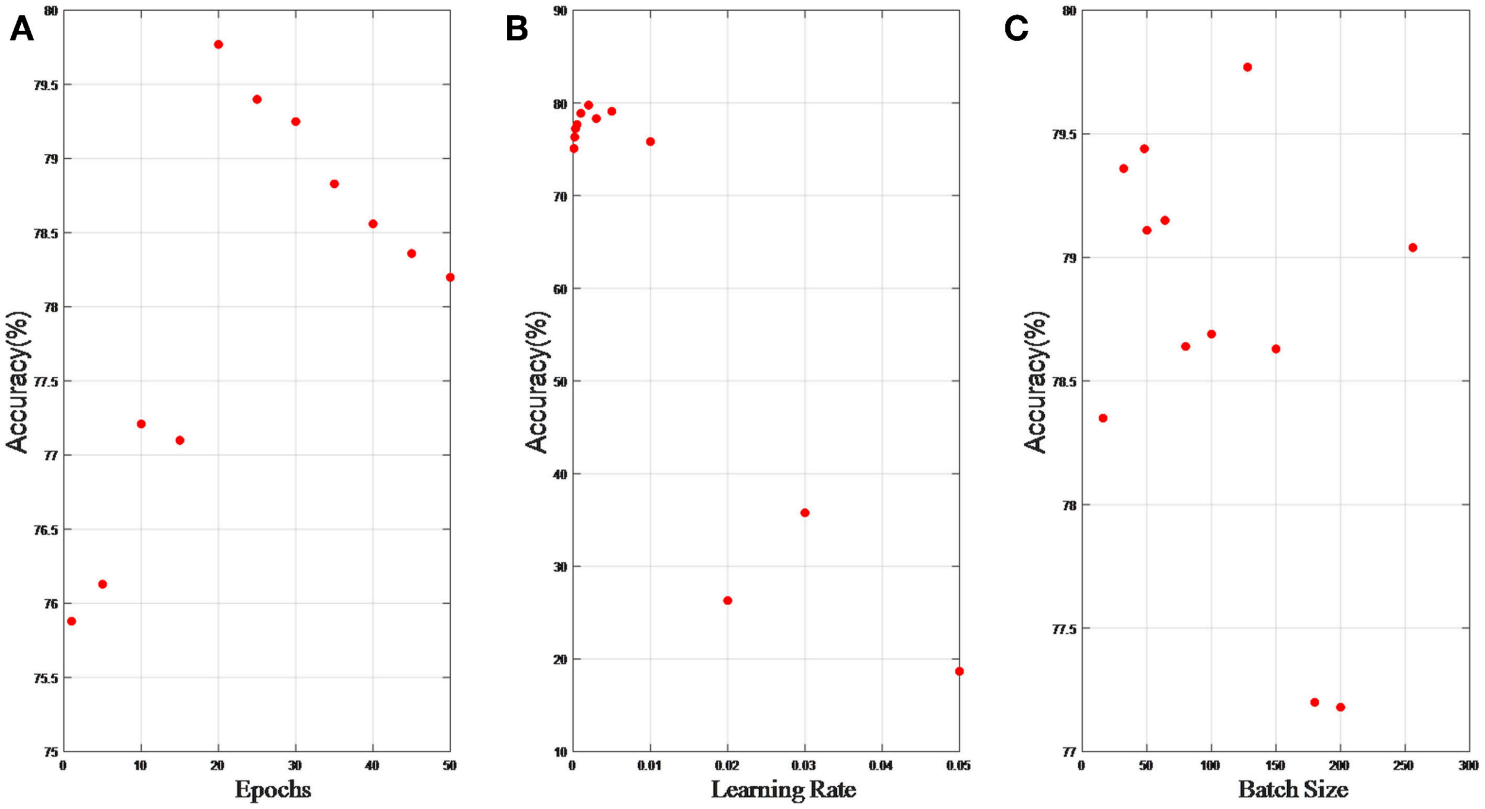
**(A)** relationship between accuracy and epochs; **(B)** relationship between accuracy and learning rate; **(C)** relationship between accuracy and batch size.

**Figure 7:**
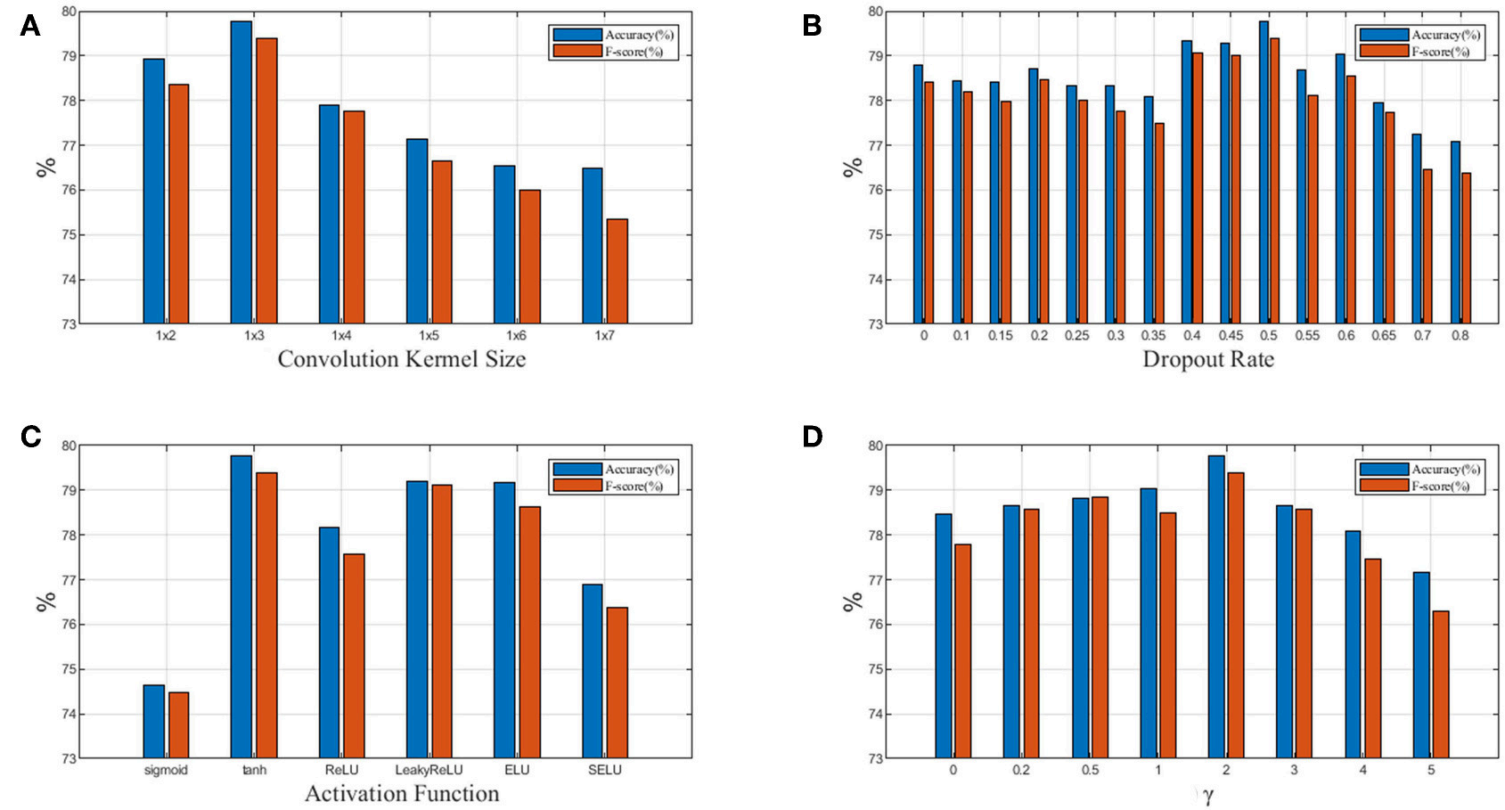
**(A)** Relationship between performance and convolution kernel size; **(B)** Relationship between performance and dropout rate; **(C)** Relationship between performance and activation function; **(D)** Relationship between performance and focusing parameter γ in focal loss. Blue denotes accuracy and red denotes*F*1_*weighted*_.

In this section, we present results of hyper parameter selection in both Figures 6, 7. Figure 6A shows how accuracy changes with epochs, and epochs are set 1, 5, 10, 15, 20, 25, 30, 40, 50, and 100, respectively in experiments. When epochs is above certain epochs like 20, the performance of the LP-GroupNet actually decreases drastically due to over-fitting. It is evident that the LP-GroupNet achieves the best performance when the epochs is 20 as you can see from Figure 6A.

Figure 6B shows the relationship between accuracy with learning rate, respectively. We set the learning rate 0.05, 0.03, 0.02, 0.01, 0.005, 0.003, 0.002, 0.001, 0.0005, 0.0003, 0.0002, and 0.0001, respectively in the experiments. It is clear that the LP-GroupNet obtains the best performance when the learning rate is 0.002 according to Figure 6B.

Figure 6C shows how batch size affects the LP-GroupNet performance, and we set batch size to 16, 32, 48, 50, 64, 80, 100, 128, 150, 180, 200, and 256, respectively. Accuracy changes with batch size quite significantly as you can see from Figure 6C. Results from the experiments show that the GroupNet achieves the best performance when batch size reaches 128.

In Figure 7A, we test 6 different convolution kernel sizes. The LP-GroupNet achieves best performance when convolution kernel size is 1 × 3. Furthermore, we also conclude that smaller convolution kernel works better than larger convolution kernel in previous works. Figure 7B presents how dropout rates influence the classification performance. It is shown that the LP-GroupNet gets the better performance when dropout rate is 0.5. It is difficult to find considerate dropout rate in the experiments as you can see from Figure 7B, because there is not a good way to find the best dropout rate theoretically except by experiments.

Figure 7C shows a performance comparison among six different activation functions: tanh, sigmoid, ReLU, LeakyReLU, ELU, and SELU. The tanh receives the best performance with 79.77% based on the GroupNet, while sigmoid receives the worst performance with 74.65%. It is noticeable that LeakyReLU and ELU both get accuracy over 79%. In order to achieve considerable performance, the tanh function is more adaptive as activation function than others in this work. Figure 7D shows how focusing parameter γ in focal loss affects the LP-GroupNet performance and γ is set 0, 0.2, 0.5, 1.0, 2.0, 3.0, 4.0, and 5.0, respectively. When focusing parameter is 0, focal loss is equivalent to standard cross entropy loss. It is clear that it results in the best performance with the accuracy of 79.77% when focusing parameter γ is 2.

Table 1 gives a comparison between Adam optimizer and SGD optimizer. It is apparent that Adam optimizer outperforms SGD optimizer. Furthermore, SGD optimizer requires 160 epochs to achieve the accuracy at 75.09%, while Adam optimizer uses 20 epochs to achieve the accuracy 79.77%. With trading-off on training time and accuracy, Adam is selected as optimizer.

**Table 1:** Comparison of Adam and SGD.

**Optimizer**	**Accuracy (%)**	***Precision*_*weighted*_ (%)**	***Recall*_*weighted*_ (%)**	***F*1_*weighted*_ (%)**
SGD	75.09	74.50	75.09	74.50
Adam	79.77	79.84	79.77	79.40

Table 2 presents the results for LP-GroupNet, LP-GroupNet-3, and LP-GroupNet-4. The results illuminate that the LP-GroupNet gets better performance than LP-GroupNet-3 and LP-GroupNet-4 models. It confirms that when the number of partition convolution units is 2 in group block, the GroupNet is able to handle the data more effectively and achieves the performance as we expected.

**Table 2:** Comparison of different number of partition convolution units in group block.

**Model**	**Accuracy (%)**	***Precision*_*weighted*_ (%)**	***Recall*_*weighted*_ (%)**	***F*1_*weighted*_ (%)**
LP-GroupNet	79.77	79.84	79.77	79.40
GroupNet-3	79.66	79.42	79.66	79.22
GroupNet-4	79.20	78.88	78.20	78.88

Table 3 lists the final optimal hyper-parameter settings.

**Table 3:** Hyper-parameter settings of the GroupNet.

**Hyper-parameter**	**Setting**
Learning rate	0.002
Epochs	20
Batch size	128
Convolution kernel size	1 × 3
Dropout rate	0.5
Activation Function	tanh
γ	2
Optimizer	Adam
The number of partition convolution units	2

### Comparison of Different Methods

Table 4 presents comparison results of the GroupNet and other CNN models based on LP method. The GroupNet achieves the best performance and increases 1.21% at least than other four CNN models on all evaluation measures.

**Table 4:** Comparison of CNN models based on LP method.

**Model**	**Accuracy (%)**	***Precision*_*weighted*_ (%)**	***Recall*_*weighted*_ (%)**	***F*1_*weighted*_ (%)**
GroupNet	79.77	79.84	79.77	79.40
IGCNet	78.08	77.64	78.08	77.65
GoogleNet	78.56	79.02	78.56	78.41
AlexNet	76.28	77.03	76.28	76.10
VGGNet	78.17	77.79	78.17	77.46

It is observed that the BR-GroupNet model provides the accuracy with 80.54% in Table 5. It increases over 0.77% than LP-GroupNet on all evaluation measures and *F*1_*weighted*_ receives the best improvement with 0.95%, which demonstrates that BR-GroupNet model is more suitable for this work than LP-GroupNet.

**Table 5:** Comparison of LP-GroupNet and BR-GroupNet.

**Model**	**Accuracy (%)**	***Precision*_*weighted*_ (%)**	***Recall*_*weighted*_ (%)**	***F*1_*weighted*_ (%)**
LP-GroupNet	79.77	79.84	79.77	79.40
BR-GroupNet	80.54	80.70	80.54	80.35

Table 6 presents a comparison among correlated loss and other loss functions based on the BR-GroupNet architecture. For convenience, cross entropy loss is named as CE in short, focal loss as FL, correlated loss based on cross entropy loss as CL1, and correlated loss based on focal loss as CL2.

**Table 6:** Comparison of different loss functions based on the BR-GroupNet.

**Loss**	**Accuracy (%)**	***Precision*_*weighted*_ (%)**	***Recall*_*weighted*_ (%)**	***F*1_*weighted*_ (%)**
CE	79.05	78.77	79.05	78.54
FL	80.54	80.70	80.54	80.35
CL1	79.66	80.59	79.66	79.30
CL2	81.13	81.37	81.13	81.02

It is obvious that CL2 gets the best accuracy with 81.13%. The results also demonstrate that CL works better than FL and CE based on the BR-GroupNet in this work, which increases approximately 0.6% on all metrics. The results from CL1 and CL2 demonstrate that correlation coefficient value between two chronic diseases is selected as hyper parameter of CL can work as we expected. Furthermore, FL achieves better performance than CE, which confirms that FL can improve classification performance by reducing the proportion of correctly classified instance loss in all loss in the training phase.

Table 7 presents the results for the BR-GroupNet-CL, four state-of-art CNN architectures, two RNN architectures (LSTM and GRU) and seven classical machine learning methods. According to these results, deep learning methods get better performance than classic machine learning methods generally, which show deep learning methods have great potentials in disease prediction. It is apparent that the BR-GroupNet-CL architecture provides the best performance among all of them on all metrics, while the SVM receives the worst performance. IGCNet, GoogleNet, AlexNet, VGGNet, LSTM, GRU, and BPMLL show similar performance and they all receive over 75% on all evaluation measures. According to the Table 7, BR-GroupNet-CL gets the best accuracy and *F*1_*weighted*_ with 81.13 and 81.02%, respectively, and it increases 2.61% than other comparative methods which confirms that the proposed BR-GroupNet-CL is more able to receive considerable performance for multi-label chronic disease classification. Particularly, BR-GroupNet-CL model achieves Re*call*_*weighted*_ with 81.13% and increases at least 2.57% comparing to other methods, which is a considerable improvement for disease classification clinically.

**Table 7:** Comparison of GroupNet model and other comparative methods.

**Model**	**Accuracy (%)**	***Precision*_*weighted*_ (%)**	***Recall*_*weighted*_ (%)**	***F*1_*weighted*_ (%)**
BR-GroupNet-CL	81.13	81.37	81.13	81.02
IGCNet	78.08	77.64	78.08	77.65
GoogleNet	78.56	79.02	78.56	78.41
AlexNet	76.28	77.03	76.28	76.10
VGGNet	78.17	77.79	78.17	77.46
DNN	71.10	75.70	71.12	72.61
LSTM	75.83	75.31	75.83	75.24
GRU	76.35	76.34	76.35	75.58
DT	77.26	77.12	77.34	77.12
MLP	74.94	74.40	74.95	74.40
SVM	48.89	42.2	49.91	41.6
SMO	70.12	67.60	70.12	67.42
ML-KNN	51.03	60.21	53.02	50.47
BPMLL	76.65	76.72	76.65	76.32

## Conclusions

We propose a novel group block inspired by group convolution strategy to reduce the number of convolution parameters and improve the classification performance. Furthermore, we develop the GroupNet based on group block, then combine GroupNet with BR and LP methods for multi-label classification of chronic diseases, respectively. We present a cost sensitive learning algorithm named correlated loss to improve the performance. The results indicate that the proposed GroupNet gets the best accuracy with 81.13%, which is nearly 2.6% higher than all other comparison methods.

In the future work, we will focus on enhancing the learning ability of the CNN model and reduce over-fitting in the training. The transfer learning and adversarial learning methods will be applied to the model.

## Author Contributions

RL conceived the project. XZ implemented the algorithm and performed the computational analysis. SZ and HZ supervised the experiments. XZ, RL, SZ, and HZ drafted the manuscript. All authors revised and approved the final version of the manuscript.

### Conflict of Interest Statement

The authors declare that the research was conducted in the absence of any commercial or financial relationships that could be construed as a potential conflict of interest.
